# Requirements and challenges for hybrid intelligence: A case-study in education

**DOI:** 10.3389/frai.2022.891630

**Published:** 2022-08-08

**Authors:** Bert Bredeweg, Marco Kragten

**Affiliations:** ^1^Faculty of Education, Amsterdam University of Applied Sciences, Amsterdam, Netherlands; ^2^Informatics Institute, Faculty of Science, University of Amsterdam, Amsterdam, Netherlands

**Keywords:** Qualitative Reasoning, science education, systems thinking with qualitative representations, real-world application problems, hybrid human-AI systems

## Abstract

The potential for Artificial Intelligence is widely proclaimed. Yet, in everyday educational settings the use of this technology is limited. Particularly, if we consider smart systems that actually interact with learners in a knowledgeable way and as such support the learning process. It illustrates the fact that teaching professionally is a complex challenge that is beyond the capabilities of current autonomous robots. On the other hand, dedicated forms of Artificial Intelligence can be very good at certain things. For example, computers are excellent chess players and automated route planners easily outperform humans. To deploy this potential, experts argue for a hybrid approach in which humans and smart systems collaboratively accomplish goals. How to realize this for education? What does it entail in practice? In this contribution, we investigate the idea of a hybrid approach in secondary education. As a case-study, we focus on learners acquiring systems thinking skills and our recently for this purpose developed pedagogical approach. Particularly, we discuss the kind of Artificial Intelligence that is needed in this situation, as well as which tasks the software can perform well and which tasks are better, or necessarily, left with the teacher.

## Introduction

The expected added value of Artificial Intelligence was already high at its inception (McCarthy et al., 1955). Meanwhile, impressive results have been obtained, but these solutions are typically highly specialized (e.g., Silver et al., 2016). The realization of Artificial General Intelligence (AGI) or strong Artificial Intelligence (e.g., Kurzweil, 2005) has not yet happened, and it may take a long time for it to happen (Marcus and Davis, 2019). Instead of aiming for AGI, the idea of Hybrid Intelligence is being proposed (Akata et al., 2020). Hybrid Intelligence combines human intelligence with machine intelligence, with the goal of augmenting human capabilities as opposed to replacing them, while simultaneously harvesting the potential of smart machines.

In the area of Intelligent Tutoring Systems, which was traditionally highly focused on automating tutoring to the max (Wenger, 1987), such alternative hybrid approaches are also discussed. Chou et al. (2011) report a study in which two virtual teaching assistants successfully aid the teacher. One assistant focuses on evaluating student's answers and the other on generating hints. Baker (2016) argues that successful automated tutoring systems do not show general (teaching) intelligence, but rather excel in a specific capability. As such, they emphasize the use of educational data mining to support human-decision-making. Another example of a hybrid approach is the work of Paiva and Bittencourt (2020) who implemented an authoring tool that deals with educational data from an online course to support instructors in making pedagogical decisions. Holstein et al. (2019) report on a study that investigates students and teachers needs with regard to human vs. Artificial Intelligence instruction help-signaling and help-giving. They found that teachers desire greater real-time support from the automated tutors, and that students emphasize their need for help-signaling without losing face to peers. Holstein et al. (2020) present a framework consisting of a set of dimensions that describe how hybrid teacher/AI adaptivity can augment performance and enhance co-learning on instructional goals, relevant information, instructional actions and decisions.

The examples show that the concept of Hybrid Intelligence in education is being discovered. Additional studies and real-life applications may help to further understand and develop this approach. In this contribution, we report on a case study that uses smart tutoring software in secondary education. While Intelligent Tutoring examples often focus on problem solving, we focus on learning by creating qualitative representations. Learners learn systems thinking by creating a diagram that captures a causal understanding of how a system works. Different from typical problem assignments, in which case the solution amounts to a specific answer such as a number after having performed the required calculations, learners create and deliver a structure consisting of a set of ingredients and relationships among these (Spitz et al., 2021a).

The organization of this paper is as follows. Section ‘What makes a system Artificial Intelligent?’ briefly reviews the field of Artificial Intelligence research in order to define what we mean when we refer to an Artificial Intelligence system. Next, we move to the case study in which learners in secondary education acquire systems thinking skills and the hybrid teacher-software arrangement to support that. Section ‘The case-study: An automated intelligent systems thinker in secondary education’ describes our recently developed intelligent tutoring system and the accompanying pedagogical approach that supports learners in creating their cause-and-effect diagrams. Section ‘Teacher's role’ discusses the role of the teacher and how it complements and intertwines with the actions of the tutoring system. Section ‘Conclusions and discussion’ concludes this contribution and Section Future work highlights directions for future research.

## What makes a system Artificial Intelligent?

It remains intriguing to observe computers solve problems that up to then only people could solve well. Even more when the computer solves versions of those problems that it has not been given explicitly before. On the other hand, the ubiquitous pocket calculator is generally not discussed as an example of smart software, even though it outperforms most humans when it comes to doing mathematics. What is it that characterizes Artificial Intelligence since it came into existence in the 60s?

### The reoccurring trinity

Let us start with a short historical perspective. One inspiration for Artificial Intelligence originates from Psychology. When the cognitivist paradigm (Lindsay and Norman, 1977) succeeded the behaviorist paradigm (Skinner, 1974), computer programs became fashionable as cognitive or mental models (Gentner and Stevens, 1983). A requirement for developing cognitive models is to make the solution generic. Instead of being able to solve one specific example, a viable solution is capable of solving all possible instances of the problem.

Over the years, many ingenious algorithms have been invented (Bratko, 2012). Additionally, the importance of adequate representations became recognized, both the formal language (the knowledge representation language) and the representation of substantive knowledge in it (the knowledge base). The endeavor grew into automating miscellaneous kinds of human expertise such as the expertise of chess players, physicians, designers, etc. (Schreiber et al., 1993). A noteworthy milestone was reached in 1987 when IBM's Deep Blue II program successfully defeated Kasparov, the then reigning chess grandmaster. Notice that, improved hardware was also a key enabler for this milestone (BNVKI, 2021).

**Figure 1:**
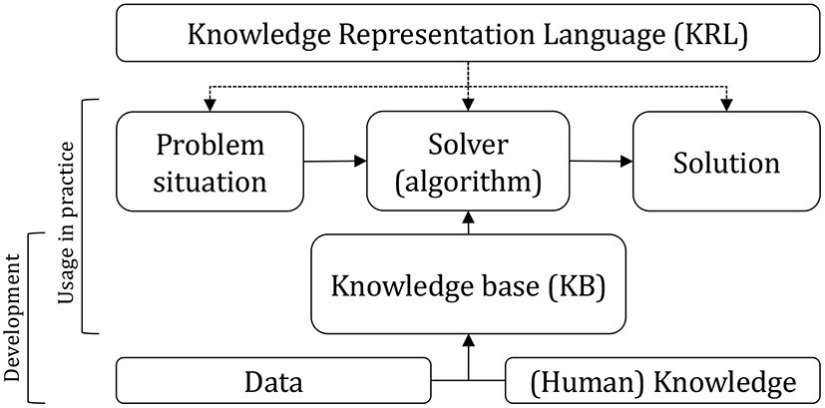
AI system characteristics. When developed, knowledge or data is obtained from humans and/or data resources, processed and stored into a Knowledge Base (KB). The KB adheres to a Knowledge Representation Language (KLR) which is also the basis for the development of the solver. When used in practice the solver receives a case (problem situation), and deploys the KB to develop a solution.

The wealth of ideas and approaches is enormous (van Harmelen et al., 2008) and the area is still advancing (Moschoyiannis et al., 2021). This also holds for the work on cognitive systems (Nirenburg, 2017). If, in hindsight, we consider the overarching research agenda, it becomes apparent that Artificial Intelligence works on three key questions (see also Figure 1):

- How to represent? The focus here is on the development of (semi-)formal languages, typically referred to as a *Knowledge Representation Language (KRL)*. Essentially a set of interrelated concept types that together conform to a certain semantics and that can be used to store (or represent) pieces of information.^1^
- What to represent? The process of selecting a certain amount of knowledge (or information), untangling it into elementary parts in accordance with the KRL and storing it. The process can be executed by humans, but also (partly) automated using software. The result is typically referred to as the *Knowledge Base (KB)*.
- How to reason? The development of *solvers* or *algorithms*, often tailored toward the specifics of the KRL, and their deployment to solve problems. Concerning the latter, the algorithm obtains or receives information about an actual case or *problem situation* and is able to draw conclusions (*solution*) by relating this input to the KB and making the appropriate inferences.

Depending on the actual implementation the appearance and use of an Artificial Intelligence system can be highly different. For instance, an automated agent continuously regulating some system as opposed to a classifier that each runtime produces a particular output.

Neural networks are also among the early ideas researched within the context of Artificial Intelligence (McCullogh and Pitts, 1943; Rosenblatt, 1958). With the arrival of abundant data and significant faster hardware, neural networks are now also well developed (LeCun et al., 2015; Goodfellow et al., 2016). They received much attention since the computer won the game of Go (Silver et al., 2016). Although the proclaimed potential is also critically reviewed (Marcus and Davis, 2019). Neural networks also adhere to the above described trinity: (i) there is a representation language consisting of interconnected units (referred to as neurons, layers, etc.), (ii) there is a body of information stored using this representation (typically, build from a huge set of examples), and (iii) there is an algorithm that reasons about specific cases using this stored information. The creation of the stored information, the “knowledge base”, can be automated in the case of isolated, formal contexts (Silver et al., 2017). However, for real applications the organization of data (data wrangling) is a complex and time-consuming task, typically performed by human experts (e.g., Kuhn and Johnson, 2019).

### Truly intelligent?

As discussed above, systems referred as to Artificial Intelligence concern three intertwined components: the representation language, the stored content, and the reasoning. When these components are well established, an artificial system can be deployed in the real-world situation for which it was developed, where it will behave according to its capacity.

A number of concerns associated with intelligent behavior are often brought up when (thinking about) using Artificial Intelligence in practice (e.g., Marcus and Davis, 2019; Aicardi et al., 2020):

- Specialization. It is generally known that Artificial Intelligence systems are highly specialized (or limited, if one prefers) and only work well for the specifics they were developed for. A system aiding physicians in finding deviating spots in x-rays, will do exactly that, and nothing else.
- Reliability and trustworthiness. Exactly when will the system fail? Is it capable of handling all the potential cases correctly? Can the software be trusted? Will it behave ethically? Notice that, the software itself typically has no clue regarding its own competence and actions, nor its limitations.
- Transparency and explanation. Can the software explain its reasoning? Explain how it came to a certain result? Moreover, can the software argue why a result or conclusion is correct or viable? In fact, the dichotomy between effective reasoning vs. insightful explanations thereof, is a long standing challenge in Artificial Intelligence.

Does having these limitations make an Artificial Intelligence less smart? Do humans not have similar limitations? Why do we want to regard human-made computer software as intelligent in the first place? These are difficult questions to answer. In fact, the answers depend on the perspective taken. The categorization of Artificial Intelligence as put forward by Russell and Peter Norvig (2020) is helpful in this respect. Instead of emphasizing a particular technology or a characteristic of intelligence, their focus is on the reference to which the solution is compared. Consequently, multiple kinds of Artificial Intelligence research and applications exist:

- Thinking humanly: The cognitive modeling approach.
- Acting humanly: The Turing test approach.
- Thinking rationally: The “laws of thought” approach.
- Acting rationally: The rational agent approach.

Table 1 shows examples to further illustrate this framework. Consider the pocket calculator mentioned earlier. We can argue that it “thinks” fully rational, following the rules of mathematics. As such, we should acknowledge that it implements a form of intelligence, even though it is not considered a typical Artificial Intelligence system (among others, it misses the knowledge base component discussed above). For any cognitive system (a system oriented toward human skills and capabilities) it should at least act humanly (e.g., pass the Turing test) and dependent on the (research) goal possible also think humanly. For an application of Artificial Intelligence supporting a physician in doing medical diagnosis we would definitely require a fully rational thinking machine. If the robot is also expected to interact with patients, maybe it should also have features of acting humanly. If it is also expected to be a pro-active and caretaking system, an autonomous robot that acts rationally is probably wanted. Similar arguments hold for intelligent applications in education. Foremost, it should be a rational thinking machine that is capable of handling the subject matter in interaction with learners. If it is also expected to be pro-active in the class, or even take a leading role, an autonomous rationally acting robot will be needed. Should it also act humanly? Maybe, but such behavior may also hamper optimal teaching behavior. After all, typical human behavior, even that of experts, may not always be the best solution in a challenging situation (Holstein et al., 2019).

**Table 1:** Which intelligence do Artificial Intelligence systems need?

	**Pocket calculator**	**Cognitive system**	**Medical diagnosis**	**Education**
Thinking humanly		?		
Acting humanly		x	?	?
Thinking rationally	x		x	x
Acting rationally			?	?

## The case-study: An automated intelligent systems thinker in secondary education

Let us now discuss an Artificial Intelligence system in education. As a case-study, we focus on learners acquiring systems thinking skills. Systems thinking is an important skill for humans to master (e.g., NGSS, 2013), but difficult to learn (e.g., Sweeney and Sterman, 2007). We develop and investigate a new pedagogical approach to having learners in secondary education acquire this skill using qualitative representations (https://denker.nu/). The approach covers K8-12 and is linked to the curriculum in the subjects of biology, physics, geography and economics. Table 2 gives an overview of the main tasks involved and the distribution among the participants (including the intelligent software, AI-App).

**Table 2:** Summarizing overview of tasks, including task description, the executing agent, the resources used to accomplish the task, the output the task delivers, and the beneficiary who uses the output.

**Task description**	**Agent**	**Resources**	**Output**	**Beneficiary**
Creating a knowledge-base for a curriculum topic	Teacher	Curriculum	KB-norm	AI-App
Creating a workbook	Teacher	Curriculum; KB-norm; AI-App	Workbook	Learner
Engaging in a dialogue to address a knowledge deficiency	Teacher	Learner request; KB-learner; AI-App; Expert knowledge	Advanced explanation	Learner
Managing the classroom and engaging learners	Teacher	Class behavior and history; Learner characteristics	Effective learning environment	Learner
Learning by creating a representation	Learner	Workbook; AI-App	KB-learner	AI-App; Teacher
Calling AI-App to compute system behavior for KB-learner	Learner	KB-learner; AI-App	Inferred system behavior	Learner
Asking for help on a knowledge deficiency	Learner	Workbook; KB-learner; Inferred system behavior	Learner request	Teacher
Finding deviating ingredients in KB-learner (norm-based cueing)	AI-App	KB-norm; KB-learner	Discrepancies highlighted	Learner
Typing deviating ingredients in KB-learner (norm-based advice)	AI-App	KB-norm; KB-learner; Error type recognizer	Discrepancies error-typed	Learner
Identifying and summarizing correct, incorrect and missing ingredients in KB-learner	AI-App	KB-norm; KB-learner; Discrepancies	Progress bar	Learner
Finding discrepancies in initial settings when calling AI-App	AI-App	KB-learner; Initial settings requirements	Advice on problem situation	Learner
Finding feedback-loop in KB-learner	AI-App	KB-learner; Feedback-loop recognizer	Feedback-loops highlighted	Learner
Describing and predicting learners' learning behavior	AI-App	KB-learner; Action-Log; Automated statistics	Overview of learners' learning behavior	Teacher

*KB refers to the representations (knowledge-base) created by the teacher (KB-norm) and by the learner (KB-learner). AI-App refers to the set of algorithms implemented in the AI software*.

For education the goal is to create smart people and the Artificial Intelligence is used as a tool to enable that. There are at least two reasons why qualitative representations form an interesting set of intelligent tools for education. Firstly, as with any representation, when used by people representations strongly steer the development of knowledge and insights (Davis et al., 1993). As such, having learners construct representations is a valuable pedagogical instrument for implementing active learning (Prain and Tytler, 2012). Secondly, the Qualitative Reasoning community particularly focused on explicating the implicit knowledge considered essential for reasoning about the behavior of (physical) systems. This resulted in an explicit vocabulary underpinning automated reasoning. In fact, the community developed an explicit ontology (Liem, 2013) for (automated) systems thinking. Modern educators emphasize the importance and challenge of supporting learners in lower and upper secondary education in acquiring systems thinking skills (Jacobson and Wilensky, 2006; Ben-Zvi-Assaraf and Orion, 2010; Curriculum.nu, 2021). Qualitative representations can be deployed for this purpose. Their suitability is even more profound because of the accompanying automated reasoners, which makes them outstanding candidates for intelligent interactive tools for learning systems thinking.

Our approach is based on a classroom situation with on average 30 learners and a teacher. Additionally, it includes an intelligent software for creating qualitative representations and a workbook to guide learners and teachers during this process. The role the software, particularly in relation to the learner, is described below. Section ‘Teacher's role’ describes the role of the teacher.

### Knowledge representation language and reasoning (solver)

The software implements an automated intelligent systems thinker (Bredeweg et al., 2009). It builds on research from Artificial Intelligence known as Qualitative Reasoning (Weld and de Kleer, 1990; Forbus, 2018). The KRL consist of ~15 concepts to describe dynamic systems, including notions such as entity, quantity, value, change, causality, in/equality, etc. The KRL is logic-based and does not use any numerical information. The main reasoning task of the solver is prediction of system's behavior, which includes a whole range of specific algorithms implementing subtasks, such as influence resolution, inequality reasoning, reasoning with assumptions, reasoning with inheritance, etc.

To be an effective tool for learning, it is important to acknowledge that systems thinking is a complex skill. It requires an approach in which the skill is gradually build up. From that perspective it is relevant to realize that the subject matter currently taught in secondary education is also complex and learned stepwise across multiple years as specified by the curricula. In accordance with these constraints, the automated systems thinker is organized such that it is able to work at distinct levels of complexity. There are five levels in total, roughly corresponding to the complexity needed in grade 8–12 (Bredeweg et l., 2010).

### Knowledge base

**Figure 2:**
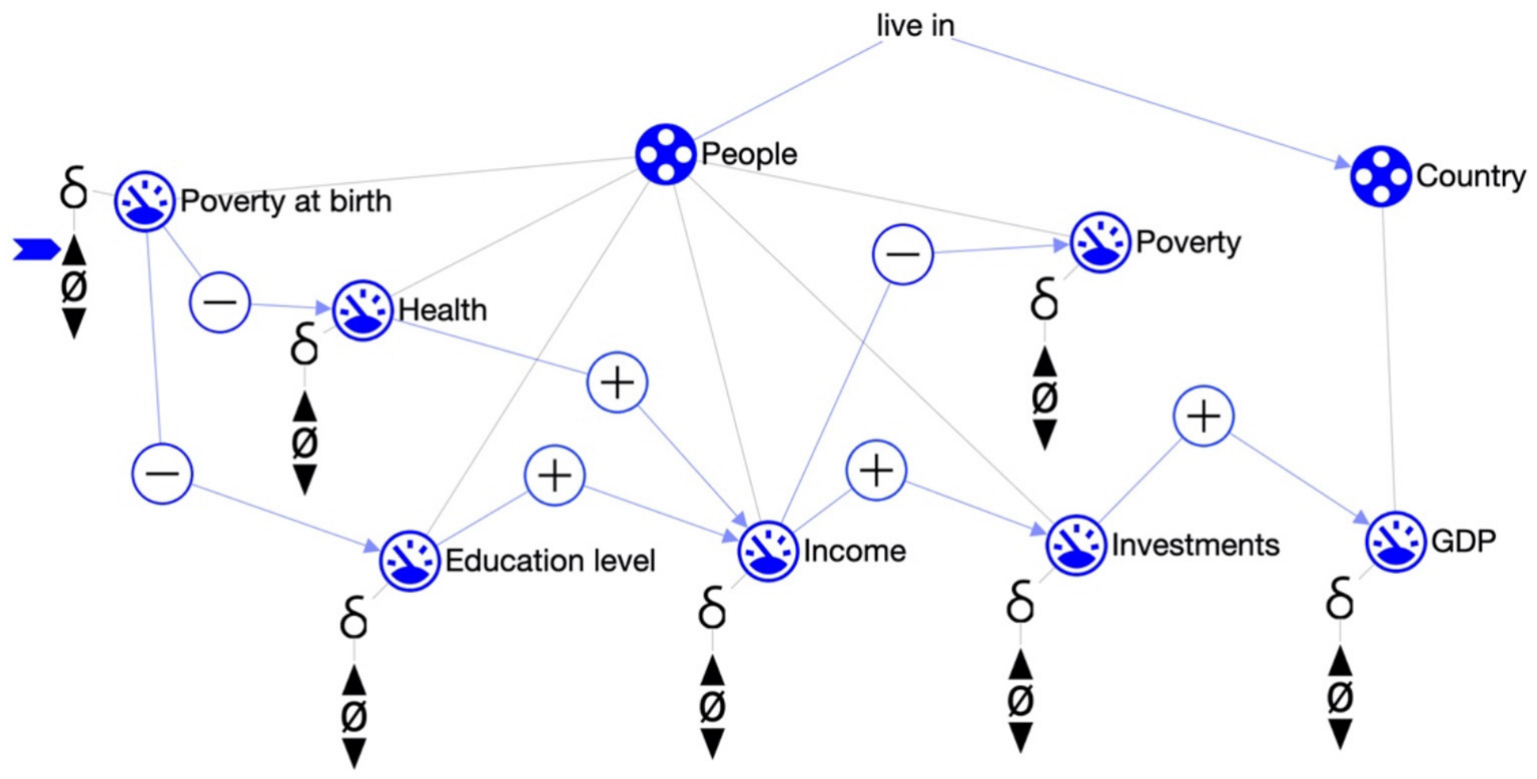
Poverty (Spitz et al., 2021b). *Entities* are *People* and *Country*, whose *relationship* is *live in*. There are seven *quantities*, such as *health* and *income*. Quantities are related by positive (+) and negative (–) *causal* relationships. The direction of change is denoted by the δ. In this case, it is only specified for *poverty at birth*, which is set to be increasing.

As discussed in Section ‘The reoccurring trinity’, a well-developed KB is a typical component of Artificial Intelligence systems. In other work, we developed such KBs (e.g., Bredeweg and Salles, 2009). However, dealing with education brings different requirements. An important insight from working with teachers has been that they have specific constraints regarding what their learners need to learn, typically following the details as specified in the curricula. For a smart tool to successfully collaborate with teachers in educating learners, this tool should be adjustable to these requirements. However, covering all the material present in the text books for all the subjects, and somehow managing that a specific part of that material gets in focus during a particular lesson, is simply not a realistic goal. Hence, we decided to take a different approach and develop small KBs. Each one is dedicated to a specific lesson and accompanying learning goals, and developed in collaborating with and as required by the teachers participating in the project (see also Section ‘Subject matter selection and preparation’). A small example is show in Figure 2 (Poverty, developed for geography in grade 8). See for more examples and details Kragten et al. (2021) and Spitz et al. (2021b).

### Supporting the learners in acquiring system thinking skills

The tool described in Sections ‘Knowledge representation language and reasoning (solver)’ and ‘Knowledge base’ (the automated systems thinker) can be given to learners to support them in their learning process. Essentially, learners learn by creating their own small “knowledge base”, mimicking the KB created by the teacher. Both the KRL and the solver are instruments that support the learner in doing so.

Notice, that the knowledge representation is shown to the learner as an **interactive diagram** (a kind of knowledge graph, similar as shown in Figure 2). After its initial design and implementation (e.g., Bouwer and Bredeweg, 2010) this diagrammatic representation has been further developed. Currently, it depicts all the ingredients present in the KRL, and also in the reasoning output, and it enables the learners to interact with these. As such, this graphical format is an important asset, because it hides low-level details and enables learners to work at the “content level”.

Having the graphical user interface, and the rest of the underlying tooling [as discussed in Section ‘Knowledge representation language and reasoning (solver)’], learners can now independently work on assignments and successfully complete these. However, learners may make mistakes and potentially learn incorrect details or get stuck in executing the assignment. Hence, the teacher has to be alert, monitor and assess the progress of the learners, and intervene where deemed necessary. Although maybe doable in small classes, it does make the teaching laborious for the teacher. To alleviate this burden, we have developed automated reasoners to further support the learner by providing just in-time feedback and to stimulate learners' self-reliance.

#### Norm-based cueing and advice

**Figure 3:**
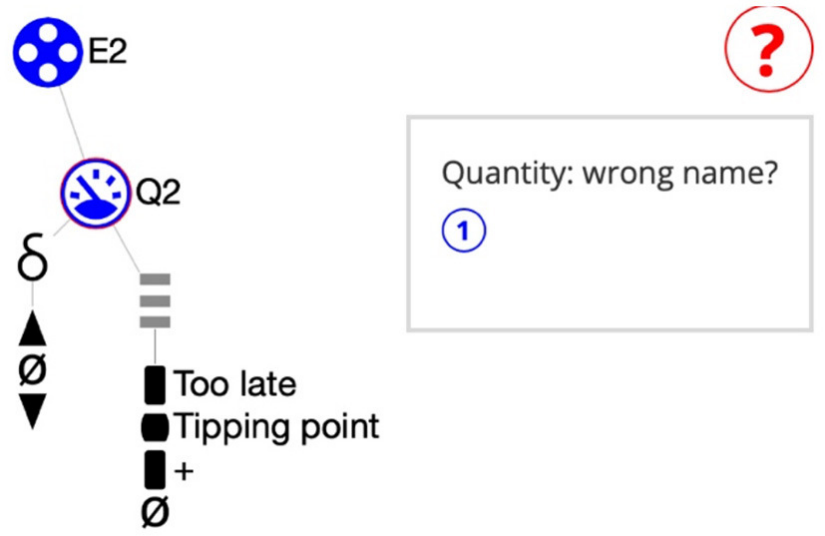
Cueing and Advice (Spitz et al., 2021a). While creating the details a quantity is wrongly named. *Cueing* highlights the erroneous ingredient (here: Q2). *Advice* suggests an error. Here: Quantity: wrong name.

The KB discussed in Section ‘Knowledge base’, which is created together with the teacher, can be used as a norm. Our current implementation compares the learner-created “knowledge-base” (KB-learner) with the KB created by the teacher (KB-norm). After each manipulation executed by the learner in the canvas a new mapping is made using a Monte-Carlo-based heuristic approach. The engine runs for at most 5 s and then returns the best mapping. Next, for each discrepancy the support provides two options for feedback. **Cueing**: a small red circle is placed around each deviating ingredient (Q2 in Figure 3) and a red question mark appears on the right-hand side in the canvas. **Advice**: when clicking on the question mark, a message-box appears showing a sentence for each deviation (in Figure 3: Quantity: Q2: wrong name?). Note that, the algorithm works domain independent, yet learners get subject specific information. For instance, whether they assign the correct quantities to each of the entities.

#### Progress bar

**Figure 4:**
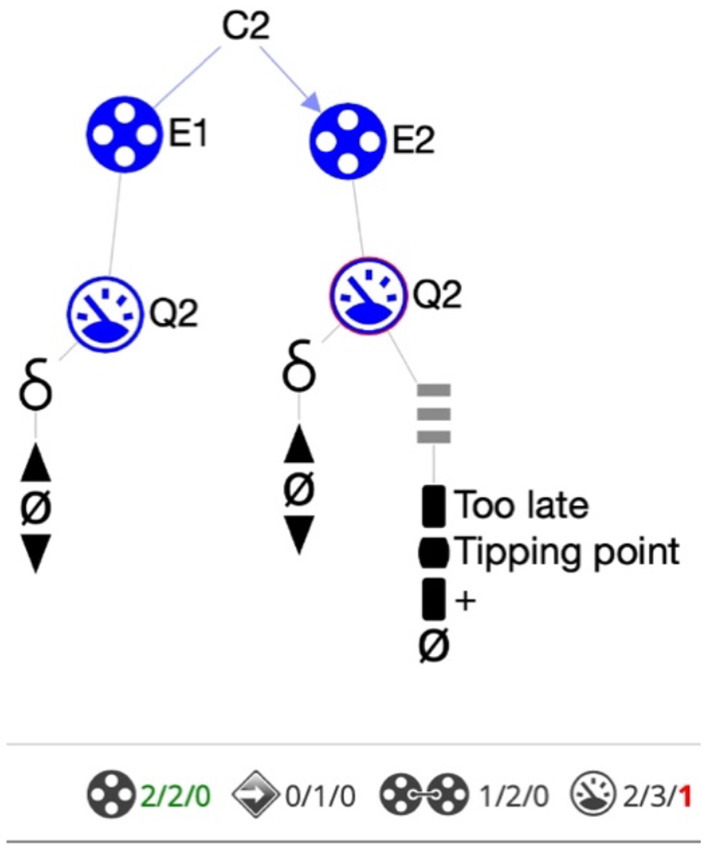
Progress bar (partly shown). The status is shown for each ingredient type at the bottom of the canvas. For instance, “Quantities 2/3/1” tells the learner that 2 quantities have been created, 3 need to be created in total and that 1 is currently incorrect (shown in red). When all ingredients have been created correctly the numbers become green, as for entities here.

Next to being informed about errors, it is also helpful for learners to get information on the degree to which they have accomplished the goals. This will support them in knowing what still needs to be done and when the goal is reached, and may also be relevant to stimulate metacognitive reflection. Our current approach implements the idea of a progress bar (Figure 4). For each ingredient *type* present in the KB-norm the bar shows (*i*) how many instances of that ingredient need to be created, (*ii*) how many at any given moment have been created, (*iii*) how many of those created are incorrect, and (*iv*) when all the details for that ingredient type are addressed (by changing font color to green). Further research is needed to find out whether this support is helpful and sufficient, without giving away too much.

Two further supports are available. The **scenario advisor** inspects the status of the problem situation when presented to the solver by the learner. If errors occur, the advisor will discover these and notify the learner. Examples are, missing initial values for quantities at the start of a causal chain and superfluous values defined for any intermediate quantity, including incorrect values that block possible outcomes from being inferred. The **feedback-loop identifier** highlights loops after the reasoning has delivered the simulation results. Two versions exist, positive feedback (change is reinforced) and negative feedback (change is reduced). The highlights are intended to help learners observe important features in the simulated system's behavior. They can also be used for coaching and further instruction.

### Analytics—Supporting the teacher

A learning analytics module has also been developed but not used in practice yet. The aim is to provide the teacher descriptive and predictive overviews, based on the progress learners make measured by the number of correct and incorrect ingredients, number of support agents calls, construction speed, etc.

## Teacher's role

Being an effective teacher is a serious challenge (Rosenshine, 2012). The key task is to create an environment that enables a group of, often diverse, learners to successfully develop their knowledge and skills. The size and complexity of this task is currently far beyond the capabilities of any automated agent based on Artificial Intelligence. However, a hybrid approach can be very effective when carefully planned and arranged, especially in specific situations. As discussed above, here we focus on lessons in systems thinking, where on average learners complete a lesson series about a specific topic in ~2 h. Which tasks does the teacher have, when using an intelligent software in this context?

### Subject matter selection and preparation

The subject matter of the lesson must be selected and prepared by the teacher. It involves (*i*) selecting learning goals for content knowledge and system thinking and thereby scoping the learning experience as a whole, (*ii*) creating a qualitative model to serve as the norm for the intelligent agent, and (*iii*) writing a small instruction workbook to guide the learners during their work (Kragten et al., 2021; Spitz et al., 2021b). If the intended lesson already exists, because it has been created and used before, the preparation becomes a simple selection step, often requiring only a few modifications of the available resources. Developing a new lesson is a more serious endeavor. Both, the construction of the workbook and the qualitative model (that is, the KB-norm) require advanced pedagogical and subject matter expertise and take a certain amount of time to create. Existing materials in terms of workbook templates and model patterns can be used to speedup this process. Templates and patterns also help to ensure quality.

### Advanced explanation

**Figure 5:**
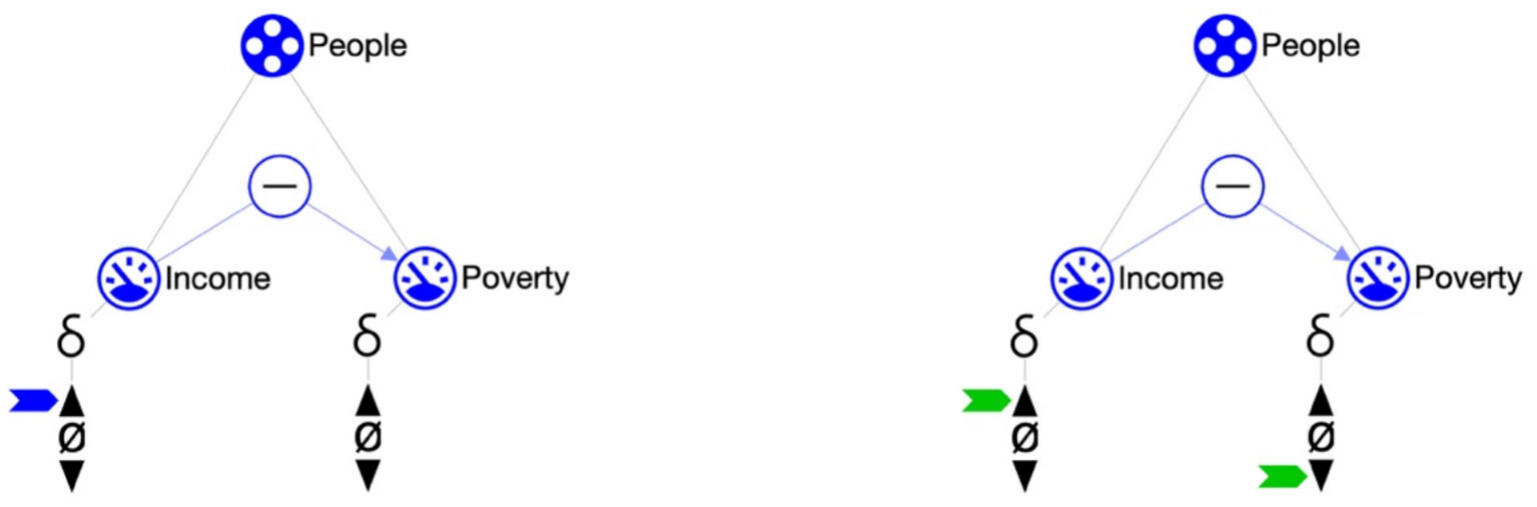
Fragment of the poverty example (Spitz et al., 2021b). The initial situation (LHS) reads as follows: Entity people has two quantities, Income and Poverty. Income is set to increase and has a negative influence on Poverty. Consequently, when simulated (RHS), poverty decreases as can be inferred from the representation.

Learners sometimes have subtle misunderstandings which are hard to overcome using logic-based explanations. For example, formal reasoning, as required by the learner when working with the qualitative representation, may get intertwined with confounding everyday concepts. This is where teachers make a significant difference. Consider the following. In a lesson on causes of poverty, a causal dependency represents the notion that an “increasing income will decrease poverty” (Figure 5). In the formal language this is represented using a negative causal dependency: the affected quantity (poverty) changes in the opposite direction of the causing quantity (income). However, in everyday conversation people typically say that “more income is good for poverty”, implying that more income will improve the situation. We have observed that most learners, possibly after some “going back and forth”, will grasp the correct interpretation. Acquiring this insight is actually a great learning experience and learners become better system thinkers. Yet, a small minority needs more advanced support that goes beyond the formal one. They often require a kind of dialogue that helps them to recognize and reflect on the misconception and guidance to revise their knowledge (Vosniadou et al., 2001). Compared to the current state of the technology a teacher is better at this task for two reasons. First, the required dialogue is advanced and often infused with specific knowledge concerning the learner involved. Second, the number of possible misunderstandings is potentially high and their kind is difficult to predict in advance. A teacher is typically more flexible and more able to address unexpected misconceptions as they occur.

### Class management and learner engagement

There is set of tasks that are concerned with class management and keeping learners engaged. Often these tasks are not specific for the subject matter at hand, yet important for the learning experience to commence and ultimately be successful. It involves tasks such as welcoming learners, inquire about their wellbeing, ensuring a positive classroom climate, inspiring them to organize their materials and start working, and probably most important, keep learners engaged throughout the learning activity. Although intelligent software can vary a lot in terms of how motivating it is for learners, the overall tasks of class management and learner engagement are beyond the scope of current technology. Real time learning analytics can support the teacher identifying students who need attention. However, making sense of the learning analytics still needs to be done by the teacher because they are best informed about their students' needs and the current situation in the classroom. Hence, these tasks remain with the teacher.

## Conclusions and discussion

Although the potential of Artificial Intelligence software is widely proclaimed, its use in education is limited. To deploy this proclaimed potential, we investigate the use of a hybrid approach in which humans and intelligence software join forces in teaching. As a case study, we focus on learners acquiring systems thinking skills in secondary education and the new pedagogical approach that we are developing for this purpose. Different from typical problem solving tasks, in which case learners produce a particular answer (e.g., a number resulting from a calculation), learners use a knowledge representation (language), and an accompanying solver, and learn by creating a small knowledge base. The latter is presented to the learners as an interactive diagram.

After briefly reviewing and clarifying our understanding of what it takes to refer to a system as being an Artificial Intelligence system, we discuss the tasks best performed by such intelligent software and which tasks are best, or necessarily, given to the teacher. The presented approach is part of ongoing research, and both used and evaluated in real educational settings. There is a clear added value to this hybrid-approach because both “agents” can now excel in the tasks they are best at, which results in improved learning (Kragten et al.^2^).

Artificial Intelligence systems typically have an extensive storage of knowledge or information which they deploy when performing the task they were developed for. We take a different approach and work with small knowledge bases, often dedicated to a particular topic aligned with the subject matter that the teacher wants the learners to work on. Taking this approach is essential. Partly, because capturing *all* the required knowledge beforehand is simply not feasible. Moreover, having a dedicated knowledge base per lesson is very helpful in fine-tuning the rest of the interaction with the learner. Notice, that the approach is still generic and that all the interaction between the software and the learners is fully automated.

Being *explicit* is an important feature of the knowledge representation (language) central to the approach presented here. Firstly, because the concepts relevant to systems thinking are all explicitly represented as unique identifiable and tangible ingredients. This makes that learners work directly with the notions relevant to systems thinking, when they create their diagram and present it to the solver. The explicitness also facilitates the automated “agents” to directly read-off relevant information and deploy this in the interaction with the learner. As such, the problem of “explainable Artificial Intelligence” does not apply here, on the contrary.

## Future work

Part of the steering during lessons in the classroom currently happens *via* the workbook. The workbook provides the learner textual information on the topic at hand, and has instructions about the steps to take. Part of the reason for having this workbook was the hypothesis that teachers prefer text-based instruments as being part of the overall setup. However, in the meantime experience in the classroom has shown that these documents create a certain amount of overhead. Teachers have requested if this can be handled in a different way. Hence, we are currently investigated whether the details provided in the workbook can also be automated, for instance based on the specifics of the knowledge base that we construct together with the teachers.

In the ongoing project, we work with a number of schools and their learners (K8-12). Each learner typically works with the approach presented multiple times per school year and over a number of consecutive years. As such, it is tempting to investigate the notion of a learner-model as a key component in the current set up. Having a learner-model would help to further tune the interaction to the specific needs of each individual student. However, the beauty of the current approach, thus without a learner-model, is that each student gets a fresh unbiased interaction each time. It is an open question whether the added value of a learner model would outweigh this benefit.

## Data availability statement

The original contributions presented in the study are included in the article/supplementary material, further inquiries can be directed to the corresponding author.

## Author contributions

All authors listed have made a substantial, direct, and intellectual contribution to the work and approved it for publication.

## Conflict of interest

The authors declare that the research was conducted in the absence of any commercial or financial relationships that could be construed as a potential conflict of interest.

## Publisher's note

All claims expressed in this article are solely those of the authors and do not necessarily represent those of their affiliated organizations, or those of the publisher, the editors and the reviewers. Any product that may be evaluated in this article, or claim that may be made by its manufacturer, is not guaranteed or endorsed by the publisher.

## Footnotes

^1^The difference between data, information and knowledge is subtle. Here we use these terms interchangeably and only make explicit distinctions when needed.

^2^Kragten, M., Spitz, L., and Bredeweg, B. *Learning Systems Thinking and Content Knowledge by Constructing Qualitative Representations in Lower Secondary Education*. (under review).
